# Factors Affecting COVID-19 Vaccine Acceptance: An International Survey among Low- and Middle-Income Countries

**DOI:** 10.3390/vaccines9050515

**Published:** 2021-05-17

**Authors:** Suzanna Awang Bono, Edlaine Faria de Moura Villela, Ching Sin Siau, Won Sun Chen, Supa Pengpid, M Tasdik Hasan, Philippe Sessou, John D. Ditekemena, Bob Omoda Amodan, Mina C. Hosseinipour, Housseini Dolo, Joseph Nelson Siewe Fodjo, Wah Yun Low, Robert Colebunders

**Affiliations:** 1 School of Social Science, Universiti Sains Malaysia, Gelugor 11800, Malaysia; suzanna.bono@usm.my; 2 Disease Control Coordination, São Paulo State Health Department, São Paulo 01246-000, Brazil; edlaine@alumni.usp.br; 3 Institute of Tropical Pathology and Public Health, Federal University of Goiás, Goiânia 74690-900, Brazil; 4 Centre for Community Health Studies (ReaCH), Faculty of Health Sciences, Universiti Kebangsaan Malaysia, Kuala Lumpur 50300, Malaysia; 5 Department of Health Science and Biostatistics, Faculty of Health, Arts and Design, Swinburne University of Technology, Hawthorn, VIC 3122, Australia; wchen@swin.edu.au; 6 ASEAN Institute for Health Development, Mahidol University, Nakhon Pathom 73170, Thailand; supa.pen@mahidol.ac.th; 7 Public Health Foundation, Bangladesh (PHF, BD), Dhaka 1217, Bangladesh; tasdikhdip@yahoo.com; 8 Department of Primary Care & Mental Health, University of Liverpool, Liverpool L69 3BX, UK; 9 Research Unit on Communicable Diseases, Polytechnic School of Abomey-Calavi, University of Abomey-Calavi, Cotonou 01 BP 526, Benin; sessouphilippe@yahoo.fr; 10 Kinshasa School of Public Health, University of Kinshasa, Kinshasa 7948, Democratic Republic of the Congo; jditekemena@hotmail.fr; 11 Uganda Public Health Fellowship Program, Loudel Towers, Level 4, Kampala, Uganda; bomoda@musph.ac.ug; 12 University of North Carolina at Chapel Hill School of Medicine, Chapel Hill, NC 27599, USA; mina_hosseinipour@med.unc.edu; 13 University of North Carolina UNC Project Malawi, Lilongwe, Malawi; 14 International Center of Excellence in Research, Faculty of Medicine and OdontoStomatology, Bamako, Mali; dolohousseini@yahoo.fr; 15 Global Health Institute, University of Antwerp, 2000 Antwerp, Belgium; josephnelson.siewefodjo@uantwerpen.be (J.N.S.F.); robert.colebunders@uantwerpen.be (R.C.); 16 Brain Research Africa Initiative (BRAIN), Yaoundé P.O. Box 25625, Cameroon; 17 Faculty of Medicine, Universiti Malaya, Kuala Lumpur 50603, Malaysia; lowwy@um.edu.my; 18 Asia Europe Institute, Universiti Malaya, Kuala Lumpur 50603, Malaysia

**Keywords:** COVID-19, vaccine acceptance, LMICs, healthcare worker, psychological distress, Brazil, Malaysia, Thailand, Bangladesh, Africa

## Abstract

Vaccination is fast becoming a key intervention against the ongoing COVID-19 pandemic. We conducted cross-sectional online surveys to investigate COVID-19 vaccine acceptance across nine Low- and Middle-Income Countries (LMICs; *N* = 10,183), assuming vaccine effectiveness at 90% and 95%. The prevalence of vaccine acceptance increased from 76.4% (90% effectiveness) to 88.8% (95% effectiveness). Considering a 90% effective vaccine, Malaysia, Thailand, Bangladesh, and five African countries (Democratic Republic of Congo, Benin, Uganda, Malawi, and Mali) had lower acceptance odds compared to Brazil. Individuals who perceived taking the vaccine as important to protect themselves had the highest acceptance odds (aOR 2.49) at 95% effectiveness.Vaccine acceptance was also positively associated with COVID-19 knowledge, worry/fear regarding COVID-19, higher income, younger age, and testing negative for COVID-19. However, chronic disease and female gender reduced the odds for vaccine acceptance. The main reasons underpinning vaccine refusal were fear of side effects (41.2%) and lack of confidence in vaccine effectiveness (15.1%). Further research is needed to identify country-specific reasons for vaccine hesitancy in order to develop mitigation strategies that would ensure high and equitable vaccination coverage across LMICs.

## 1. Introduction

In 2020, the infectious Coronavirus disease 2019 (COVID-19) had spread globally and affected individuals from all walks of life. According to the World Health Organization [1], as of 9 February 2021, the world’s cumulative COVID-19 confirmed cases added up to 106,212,882 and the total number of deaths thus far was 2,329,036. Currently, 13 COVID-19 vaccines have already been licensed by at least one country [2]. While the governments of high-income countries pre-ordered these vaccines [3], low- and middle-income countries (LMICs) may have difficulties in purchasing enough doses for their population. To bridge this gap, the COVAX initiative was created to rapidly procure and deliver doses of a safe, effective, and approved vaccine for equitable distribution around the world [4].

As vaccines are being distributed around the world, there is a debate on who should receive vaccination first [5]. The US Centers for Disease Control and Prevention (CDC) suggested that frontline healthcare workers and groups that are most at risk, such as those aged 60 and above, and persons with certain medical conditions should be prioritized [6]. This should rapidly decrease hospitalizations and deaths, allowing societies to reopen and regain a sense of normalcy. Albeit aiming for herd immunity, there are reports showing hesitancy in accepting the vaccine among diverse communities [7,8,9,10]. This has also been seen in the past, whereby vaccine acceptability has been influenced by factors such as gender [11], knowledge [12], safety and importance of the vaccine [13], and mistrust in sources relaying information about the vaccine [14,15].

As LMICs are starting to receive COVID-19 vaccines [4], it is important to understand the determinants of vaccine acceptability with the intention of creating strategies for increasing vaccine coverage in order to rapidly bring the pandemic to an end. Therefore, the current study aimed to investigate the factors affecting the acceptability of the COVID-19 vaccine in several LMICs in three different continents. We investigated the associations between the respondents’ willingness to accept the COVID-19 vaccine and different factors such as country, gender, age, educational level, working or studying in the healthcare sector, number of people in the household, area of residence, socioeconomic status, knowledge about COVID-19, worry about COVID-19, depression, anxiety, the presence of chronic diseases and comorbidities, and the perceived importance of being vaccinated. These assessments were performed assuming 90% and 95% levels of vaccine effectiveness.

## 2. Materials and Methods

### 2.1. Design and Participants

This was a descriptive cross-sectional study. Participant-inclusion criteria were to be at least 18 years old and provide informed consent to participate in this study. Ethical approval was obtained from the Ethics Committees of the participating countries (see Author Statements for details on the ethical approvals).

### 2.2. Materials

The questionnaire consisted of three sections. The first section requested participants to complete their demographic information (age, sex, country of residence, educational level, studying or working in healthcare, the (estimated) age(s) of their housemate(s), if any, self-perceived socio-economic status, and self-perceived area of residence). The second section consisted of eight questions regarding the participants’ health status, their knowledge on COVID-19, adherence to COVID-19 preventive measures, and their level of fear/worry of being infected with COVID-19. COVID-19 knowledge was evaluated by scoring the answers to the following questions (each “Yes” answer scored 1 point, and each “No” answer scored 0 points): (1) if there is a possibility of being re-infected after recovering from a previous COVID-19 infection; (2) if COVID-19 infection could be prevented by a vaccine; and (3) if there is currently an effective vaccine against COVID-19. The third section of the questionnaire consisted of four questions regarding the willingness of participants to take the COVID-19 vaccine at 90% and 95% effectiveness levels and the reasons for vaccine hesitancy.

The questionnaire also included screening tests for psychosocial disorders (anxiety and depression symptoms). The screening tests used were the Patient Health Questionnaire (PHQ-2) for depression symptoms [16] and the Generalized Anxiety Disorder (GAD-2) tool for anxiety symptoms [17]. For each scale, a score of ≥3 was considered as positive screening for the condition [16,17].

### 2.3. Procedure

The questionnaire was designed in English with the input of the investigators of the different countries, translated into the national languages of the participating countries, and pilot tested in these countries. Questionnaires were completed online using an electronic link disseminated via the social network of the investigators, using platforms such as WhatsApp, Facebook, SMS, Messenger, Twitter, Instagram, and university webpage portals between 10 December 2020 to 9 February 2021. Participation was voluntary and participants provided informed consent before attempting the questionnaire. Anonymity and confidentiality were ensured by not collecting any identifying information.

### 2.4. Weighting

A critical step prior to conducting data analyses was to ensure that all data had been weighted appropriately. For each country, the weight was estimated with the proportion of country-specific individuals in the target population (aged 15 and above within each country in 2019) [18] divided by the proportion of country-specific survey participants. This approach aimed to reduce the variations of the estimates and also to compensate the effects on the estimates due to survey over- or under-coverage [19,20].

In order to avoid having very large weights, an ad hoc rule stated in Equation (1) was used to derive the upper bound for weights [21]. No further adjustment on the weight was performed since all weight estimates were well within the upper bound.

Upper bound for weights = 5 * mean weight(1)

### 2.5. Statistical Analysis

Descriptive statistics were presented as means and standard deviations (SD) for continuous variables, and categorical variables were summarized using frequency and percentage. χ^2^ analyses were conducted to investigate reasons for vaccine hesitancy with respect to the demographic and health status variables. A series of multiple logistic regression analyses was conducted, with vaccine acceptance as the dependent variable, at both 90% and 95% effectiveness levels. The dependent variable was coded yes = 1, and no/no opinion = 0. All assumptions for multiple logistic regression were verified. The Spearman’s Rank correlation coefficient and Pearson’s correlation coefficient were used to investigate the multicollinearity of the factors. Due to the high correlation between PHQ-2 and GAD-2 scores, (ρ(10,183) = 0.74, *p* < 0.001), these variables were dichotomized into 1 = score ≥ 3, and 0 = score < 3. The variable “Importance of taking COVID-19 vaccine to protect others” was excluded from the regression models due to its high correlation with “Importance of taking COVID-19 vaccine to protect self” (ρ(10,183) = 0.86, *p* < 0.001). A p-value of less than 0.05 (two-tailed) was deemed statistically significant. All analyses were conducted using SPSS (version 27, IBM Corp, New York, NY, USA).

## 3. Results

A total of 10,491 participants completed the survey from 83 countries. Only countries with 50 participants and above were retained for analysis, which included Brazil, Malaysia, Thailand, Bangladesh, Democratic Republic of Congo, Benin, Uganda, Malawi, and Mali. After cleaning the data by removing the countries with less than 50 participants, 308 (2.9%) participants were excluded from further analysis, resulting in a total of 10,183 participants. Most of the participants were from Brazil (*n* = 6470; 63.5%), female (*n* = 6604; 64.9%), university postgraduate degree holders (*n* = 4839; 47.5%), non-healthcare workers (*n* = 6683; 65.6%), belonging to the lower middle-income category (*n* = 4459; 43.8%), and living in an urban setting (i.e., city or town; *n* = 8186; 80.4%). The mean age was 45.1 years old (SD = 15.0 years). In terms of health status, 2958 (29.0%) reported having at least one chronic disease. Regarding psychological distress, 2058 (20.2%) screened positive for depression symptoms, and 2212 (21.7%) were positive for anxiety symptoms (see Table 1 for country-specific information).

Knowledge about COVID-19 varied across countries, with low mean knowledge scores reported among participants from Benin, while Brazilian respondents reported high mean scores. Additionally, participants from Brazil also reported a high level of worry/fear of getting infected with COVID-19 (Table 2).

With regard to the participants’ willingness to take the COVID-19 vaccine when it becomes available, 76.4% (*n* = 7775) were willing to be vaccinated if the vaccine was at least 90% effective, and 88.8% (*n* = 9041) if the vaccine was at least 95% effective (χ^2^ = 544.7, *p* < 0.001). Brazil had the highest percentage of participants who were willing to be vaccinated. The most frequently endorsed reasons for vaccine refusal were the fear of vaccine side effects (41.2%) followed by a lack of confidence in vaccine effectiveness (15.1%). Malaysia had the highest percentage of participants who feared vaccine side effects (74.1%), and Thailand had the highest percentage who expressed a lack of confidence in the vaccine (44.5%). The African countries had a higher percentage of participants who endorsed the belief that the vaccine was designed to harm them (14.5–37.1%) as compared to Brazil and Asian countries (see Table 3 for country-specific information).

Group comparisons revealed that a larger proportion of males believed that the vaccine was not effective, was designed to harm them, and that they did not need the vaccine as their body was naturally strong (all *p* < 0.001). Compared to males, a greater proportion of females feared vaccine side effects (*p* = 0.001). A greater proportion of low and lower middle-income individuals were fearful of vaccine side effects, believed that the vaccine was designed to harm them, that COVID-19 did not exist, and that the vaccine was not effective (Table 4).

Two multiple logistic regression models were constructed to investigate predictors of COVID-19 vaccine willingness at different effectiveness levels (Table 5). Both models were statistically significant for 90% effectiveness (χ^2^(26) = 3772.37, *p* < 0.001; Nagelkerke R^2^ = 0.43) and 95% effectiveness (χ^2^(26) = 3400.92, *p* < 0.001; Nagelkerke R^2^ = 0.50). The two models (90% and 95% effectiveness) accounted for 43.4% and 49.5% of the variances explained by the predictors, respectively.

All countries had lower odds for COVID-19 vaccine acceptability compared to Brazil at 90% effectiveness. However, at 95% effectiveness, Thailand (aOR: 1.54, 95% CI [1.14, 2.10], *p* = 0.006) and Bangladesh (aOR: 1.43, 95% CI [1.08, 1.90], *p* = 0.012) had higher odds for vaccine acceptability. Compared to participants aged 60 years and above, those in the age groups of 18–29 years and 30–39 years had higher odds of vaccine acceptance at both effectiveness levels, especially among 18- to 29-year-olds at the 95% effectiveness level (aOR: 1.62, 95% CI [1.14, 2.28], *p* = 0.007). Females had lower odds of willingness to be vaccinated at the 95% effectiveness level (aOR: 0.75, 95% CI [0.65, 0.88], *p* < 0.001). In terms of income, those with lower-middle (aOR: 1.23, 95% CI [1.01, 1.49], *p* < 0.001, higher-middle (aOR: 1.75, 95% CI [1.42, 2.16], *p* < 0.001), and high income (aOR: 1.90, 95% CI [1.32, 2.73], *p* < 0.001) had higher odds of willingness to be vaccinated compared to those with low income at the 90% effectiveness level.

In terms of education and knowledge, participants from undergraduate and postgraduate levels had higher odds for willingness to be vaccinated compared to those who had completed primary and secondary education, particularly among undergraduate degree holders at the 95% effectiveness level (aOR: 1.50, 95% CI [1.19, 1.89], *p* = 0.001). Those who scored higher in COVID-19 knowledge had consistently higher odds of willingness to be vaccinated, particularly at the 95% effectiveness level (aOR: 2.13, 95% CI [1.96, 2.31], *p* < 0.001).

In terms of health status, participants who had tested negative for COVID-19 had higher odds of willingness to be vaccinated both at the 90% effectiveness level (aOR: 1.35, 95% CI [1.19, 1.53], *p* < 0.001) and at the 95% effectiveness level (aOR: 1.37, 95% [CI 1.15, 1.63], *p* < 0.001). The presence of at least one underlying chronic disease predicted lower odds for willingness to be vaccinated (aOR: 0.81, 95% CI [0.71, 0.92], *p* = 0.001) at the 90% effectiveness level. Participants who gave a higher rating to the importance of taking the vaccine to protect themselves had higher odds of taking the vaccine at both levels of effectiveness, particularly at the 95% effectiveness level (aOR: 2.49, 95% CI [2.34, 2.66], *p* < 0.001). Increased levels of fear/worry about being infected with COVID-19 consistently predicted higher odds of willingness to take the vaccine at 90% (aOR: 1.32, 95% CI [1.25, 1.38], *p* < 0.001) and 95% effectiveness (aOR: 1.30, 95% CI [1.20, 1.40], *p* < 0.001).

## 4. Discussion

This study aimed to examine the level of willingness to take the COVID-19 vaccine across selected LMICs, and to investigate the factors that predicted the willingness to be vaccinated at 90% and 95% vaccine effectiveness levels. We conducted the survey simultaneously across a number of LMICs and across three continents, thereby making cross-country comparisons possible.

More participants were willing to accept the COVID-19 vaccination at a higher level of effectiveness, with a significant difference of 12.4% between 90% and 95% effectiveness. Increased vaccine acceptance in relation to vaccine effectiveness was found in other studies [22,23], and was related to concerns over vaccine safety [24,25]. The overall acceptance rate of 76.4% and 88.8% for 90% and 95% effectiveness is comparable to the results of a systematic review, where the acceptance rates for most studies among the general population was ≥70% [26]. However, as a lower vaccine acceptance rate was observed among persons with low educational level, and this group was underrepresented in our sample, the true acceptance rate in the general population may be much lower.

Particularly noteworthy are the participants from Brazil, who were more likely to accept the COVID-19 vaccine compared to those in the other eight countries at the 90% effectiveness level. The higher vaccine acceptance at lower effectiveness may be due to the high COVID-19 mortality rate in Brazil. Indeed, this country has the second highest number of COVID-related deaths in the world, totaling more than a quarter million in early March 2021 [1,27]. Brazil also recorded the highest rate of transmission, with a basic reproduction number (R_0_) estimated at 2.81 [28]. This may explain why respondents from Brazil reported the highest scores for fear/worry about becoming infected with COVID-19, as well as the lowest proportions of respondents expressing fear of COVID-19 vaccine side effects and doubts about vaccine effectiveness during the by-country analysis.

The lowest vaccine acceptance was observed in the African countries. This may be related to the fact that lower COVID-19 mortality is currently observed in the participating African countries. The widespread perception that Africa is less at risk of COVID-19 has also raised questions regarding the need for major investments in vaccinations in Africa. Moreover, misinformation by the mass media [29] and perhaps a historical vaccine hesitancy in Africa, such as the polio vaccine boycott in Nigeria in the early 2000s, may have played a role [30]. Widespread online misinformation has been observed during this pandemic and could seriously threaten vaccine acceptance in countries where accurate evidence-based information is not readily accessible or where there is politicization of scientific knowledge on vaccine effectiveness and safety [29].

Participants with higher knowledge about COVID-19 had higher odds of accepting COVID-19 vaccination, and university graduates had higher odds of accepting vaccination compared to participants with primary or secondary school education. This disparity in the willingness to take COVID-19 vaccination was also found in a U.S. study, where those who did not complete high school education reported lower acceptance prevalence as compared to those who did [31]. Therefore, increasing knowledge about COVID-19, especially among those with fewer years of education, should be an effective way to increase willingness to take the vaccine.

Similar to other studies, we found less vaccine acceptance among participants with low income [22,32]. However, this is true only at the 90% effectiveness level. The lower acceptance odds of the lower income group in our study sample may be due to their lack of access to high-quality information and low health literacy [33]. In our study, individuals who self-identified as belonging to the low-income category more often believed that the vaccine is not effective. This could explain their lower acceptance odds at the 90% effectiveness level. This is reason for concern because low-income groups have been shown to be at a higher risk of contracting COVID-19 due to overcrowded living conditions, their use of public transportation, and a higher likelihood of working outside the home, all of which limit their social distancing ability [34,35]. Therefore, it is important to bridge the gap of COVID-19 vaccination willingness between individuals of lower and higher socio-economic classes.

Our study showed a similar vaccine acceptance rate between healthcare workers/students and the general population. In contrast, a study in Indonesia observed a higher acceptance rate among Indonesian healthcare workers [36], yet in the DR Congo, vaccine acceptance was lower among healthcare workers [29,30].

Attitudinal factors such as worry about COVID-19 increased the odds of accepting the vaccine. According to Wong and colleagues [37], the Health Belief Model could explain this phenomenon, whereby the perceived benefit of vaccination included a significant reduction of worry about COVID-19 infection, thus increasing worried individuals’ intention to take the vaccine. Participants who had been tested negative for COVID-19 had higher odds for willingness to take the COVID-19 vaccine. In most contexts, COVID-19 testing was mandated for individuals who had come into close contact with suspected or confirmed COVID-19 cases, or if they or their families had flu-like symptoms [38,39]. Therefore, even though they were tested negative, their awareness of the virus would be heightened by their testing experience. This may in turn lead to a higher willingness to take the vaccine.

We equally found that female respondents and participants who had at least one chronic disease had lower odds in willingness to take the vaccine. The literature on gender and COVID-19 vaccine acceptance is mixed, with most studies indicating higher male acceptance (e.g., in France, the UK, and Turkey) [40,41]; however, in a study conducted across 19 countries, a slightly higher female acceptance was reported [32]. The higher odds of vaccine willingness by males at the 95% effectiveness level may be explained by the fact that significantly more males endorsed that the vaccine was not effective, and a higher effectiveness (95%) would consequently make them more willing than females to accept the vaccine. Furthermore, the χ^2^ analysis revealed that fear of side effects was significantly higher among females. Our results are consistent with literature, whereby more females expressed concern regarding the side effects of the vaccine compared to males in a study across seven European countries [42]. In another study in Jordan and Kuwait, the belief that the COVID-19 vaccine could cause infertility was reported by 23.4% of the participants [43]. These false beliefs may lead to a lower willingness to take the vaccine. However, female-specific concerns about vaccine side effects require further investigation.

A greater proportion of younger participants aged between 18 and 39 years endorsed inaccurate beliefs about COVID-19 vaccines. This may be because this younger age group was more exposed to vaccine-related misinformation through social media. The reason why this younger age group had higher odds of vaccination acceptance requires further investigation.

Having a chronic disease lowered the odds of willingness to take the vaccine, in contrast to findings from a few developed countries such as the UK [44]. A survey in France showed that persons who reported no underlying chronic illness were more likely to refuse the vaccine [41]. Our results showed a lower vaccine acceptance among this vulnerable population at the 90% effectiveness level. However, the difference between those with and without chronic diseases disappeared at the 95% effectiveness level. The very high effectiveness level of the vaccine may have attenuated the difference in vaccine acceptance odds. The lower acceptance odds at the 90% effectiveness level may have been the consequence of negative media portrayal [41]. It needs to be noted, however, that chronic disease was self-reported, and is therefore subject to bias.

Our study suggests that reasons for vaccine refusal differ according to region. For example, participants from Asian countries (Malaysia, Thailand, and Bangladesh) recorded a high percentage for fear of COVID-19 vaccine side effects. On the other hand, the belief that the COVID-19 vaccine was designed to harm others was endorsed to a great extent in all five African countries. Therefore, interventions to increase vaccine acceptance entail targeting specific vaccine-related attitudes and knowledge pertinent to each country and cultural setting.

The strength of our online survey method was that it allowed us to very rapidly obtain information about perceptions of COVID-19 vaccination in nine low- and middle-income countries at a time when these countries were starting their COVID-19 vaccination roll out. Since then, vaccination has taken off, but only very slowly in the African countries where the population seems to be reluctant to accept the AstraZeneca vaccine, or where there were difficulties in distributing them [45]. As of 27 April 2021, only 1.6% of the total vaccine doses administered globally had been administered on the African continent [46] (Table 6).

However, several limitations of our study need to be mentioned. Due to the online approach of our survey and the self-selection of participants [47], there was an uneven distribution of participants from different countries. Given the limited access to internet, only a small number of persons from the African countries and Bangladesh participated in the survey. Therefore, weights were applied to the study data [48]. Online data collection also leaves out population segments that do not have internet access and have low literacy levels. The distribution method of the questionnaire by investigators through their social media network led to a snowballing effect. This explains the over-representation of individuals with higher education, higher socio-economic background, healthcare workers, and the urban population as it was initiated by universities that are in urban areas. Notably, in all the countries surveyed (with the exception of Brazil and Malaysia), healthcare workers/students constituted more than 50% of each country’s participants. Finally, the socio-economic and residential setting variables were self-perceived and self-estimated, and thus were subjective indicators. Online surveys should be followed up or complemented with other study designs such as in-depth qualitative explorations to understand local insights, thinking, and perceptions of the non-literate population. Future studies should use more stringent sampling methods, such as during a nationally representative household survey, specifying the balanced and adequate representation of important demographic groups [47].

This study has implications for the design of appropriate strategies that increase vaccine acceptance. Low income individuals should be targeted in all countries through community approaches by health authorities to dispel misinformation regarding COVID-19 vaccines, specifically regarding the effectiveness and side effects of vaccines. To improve COVID-19 vaccine compliance among women, vaccine safety concerns should be emphasized regarding female-specific issues such as child-bearing and fertility. As this study was conducted before the vaccine was rolled out in most countries, there is a need to conduct a similar study to examine whether vaccine acceptance has increased or decreased.

## 5. Conclusions

With the advent of mass vaccination to quell the COVID-19 pandemic, the distribution of strategically placed public health information regarding COVID-19 vaccination, delivered in locally customized and culturally appropriate language, may be instrumental in increasing the general public’s willingness to take the COVID-19 vaccine. Our study findings raise major concerns about equitable vaccination, with poorer and less educated individuals having lower acceptance. Particularly concerning is also the lower acceptance among individuals with chronic disease who most require the vaccinations. There is an urgent need to further explore and address the fears and concerns of these groups to ensure equitable access to and utilization of COVID-19 vaccines.

## Figures and Tables

**Table 1:** Participant demographics and health status (N = 10,183).

Variable	Total *n* = 10183	Brazil *n* = 6470	Malaysia *n* = 1738	Thailand *n* = 1124	Bangladesh *n* = 230	DR Congo *n* = 219	Benin *n* = 159	Uganda *n* = 107	Malawi *n* = 81	Mali *n* = 55
	*n* (%)	*n* (%)	*n* (%)	*n* (%)	*n* (%)	*n* (%)	*n* (%)	*n* (%)	*n* (%)	*n* (%)
Demographics										
Gender										
Male	3579 (35.1)	2125 (32.8)	600 (34.5)	343 (30.5)	93 (40.4)	169 (77.2)	112 (70.4)	55 (51.4)	35 (43.2)	47 (85.5)
Female	6604 (64.9)	4345 (67.2)	1138 (65.5)	781 (69.5)	137 (59.6)	50 (22.8)	47 (29.6)	52 (48.6)	46 (56.8)	8 (14.5)
Age, years										
Mean ± SD	45.06 ± 15.01	48.07 ± 14.57	41.05 ± 15.85	43.58 ± 12.76	28.62 ± 6.53	35.20 ± 8.96	28.50±10.24	33.79 ± 8.84	37.80 ± 8.63	37.44 ± 8.99
Median (Min, Max)	45 (18, 93)	49 (18, 93)	38 (18, 87)	45 (18, 81)	27 (18, 60)	35 (20, 65)	25 (18, 65)	32 (20, 63)	38 (18, 69)	36 (25, 65)
18–29	3206 (31.5)	424 (11.6)	166 (31.2)	245 (19.4)	1590 (61.3)	225 (29.7)	342 (62.3)	138 (37.4)	41 (14.7)	35 (18.4)
30–39	2707 (26.6)	734 (20.1)	116 (21.8)	205 (16.2)	891 (34.3)	308 (40.6)	121 (22.0)	138 (37.4)	114 (40.9)	80 (42.1)
40–49	1621 (15.9)	708 (19.4)	74 (13.9)	300 (23.7)	68 (2.6)	173 (22.8)	59 (10.7)	66 (17.9)	111 (39.8)	62 (32.6)
50–59	1490 (14.6)	836 (22.9)	79 (14.8)	446 (35.2)	23 (0.9)	38 (5.0)	24 (4.4)	24 (6.5)	10 (3.6)	10 (5.3)
60 and above	1157 (11.4)	941 (25.8)	97 (18.2)	70 (5.5)	23 (0.9)	14 (1.8)	3 (0.5)	3 (0.8)	3 (1.1)	3 (1.6)
Highest education level attained										
Primary/Secondary	1316 (13.0)	760 (11. 8)	328 (18.9)	177 (15.7)	23 (10.0)	7 (3.2)	11 (6.9)	3 (2.8)	6 (7.4)	1 (1.8)
Completed undergraduate degree	4028 (39.5)	2041 (31.5)	890 (51.2)	647 (57.6)	123 (53.5)	150 (68.5)	79 (49.7)	54 (50.5)	39 (48.2)	5 (9.1)
Completed postgraduate degree	4839 (47.5)	3669 (56.7)	520 (29.9)	300 (26.7)	84 (36.5)	62 (28.3)	69 (43.4)	50 (46.7)	36 (44.4)	49 (89.1)
Socio-economic category										
Low	839 (8.2)	283 (4.4)	245 (14.1)	242 (21.5)	6 (2.6)	14 (6.4)	13 (8.2)	27 (25.2)	6 (7.4)	3 (5.5)
Lower middle	4459 (43.9)	2599 (40.2)	710 (40.9)	690 (61.4)	89 (38.7)	147 (67.1)	101 (63.5)	52 (48.6)	36 (44.4)	35 (63.6)
Upper middle	4381 (43.0)	3187 (49.2)	723 (41.6)	175 (15.6)	128 (55.7)	54 (24.7)	40 (25.2)	26 (24.3)	33 (40.8)	15 (27.3)
High	504 (4.9)	401 (6.2)	60 (3.4)	17 (1.5)	7 (3.0)	4 (1.8)	5 (3.1)	2 (1.9)	6 (7.4)	2 (3.6)
Residential setting										
Rural	812 (8.0)	139 (2.1)	202 (11.6)	433 (38.5)	4 (1.7)	11 (5.1)	10 (6.3)	8 (7.5)	3 (3.7)	2 (3.7)
Suburban/Slum	1185 (11.6)	698 (10.8)	238 (13.7)	121 (10.8)	34 (14.8)	13 (5.9)	30 (18.9)	31 (29.0)	13 (16.0)	7 (12.7)
Urban	8186 (80.4)	5633 (87.1)	1298 (74.7)	570 (50.7)	192 (83.5)	195 (89.0)	119 (74.8)	68 (63.5)	65 (80.3)	46 (83.6)
Student or worker in the health sector (Yes)	3500 (34.4)	1964 (30.4)	371 (21.3)	618 (55.0)	133 (57.8)	142 (64.8)	88 (55.3)	91 (85.0)	49 (60.5)	44 (80.0)
Health status										
COVID-19 testing/Infection status										
Not tested/Does not know test results	6078 (59.7)	3283 (50.7)	1246 (71.7)	1017 (90.4)	133 (57.8)	122 (55.7)	113 (71.1)	71 (66.4)	63 (77.7)	30 (54.5)
Tested, negative	3362 (33.0)	2526 (39.1)	473 (27.2)	104 (9.3)	60 (26.1)	89 (40.6)	42 (26.4)	31 (29.0)	16 (19.8)	21 (38.2)
Tested, positive	743 (7.3)	661 (10.2)	19 (1.1)	3 (0.3)	37 (16.1)	8 (3.7)	4 (2.5)	5 (4.6)	2 (2.5)	4 (7.3)
Presence of chronic disease(s)										
Yes	2958 (29.0)	2192 (33.9)	415 (23.0)	219 (19.5)	49 (21.3)	23 (10.5)	11 (6.9)	13 (12.1)	26 (32.1)	10 (18.2)
Psychological distress										
Depression symptoms (PHQ-2 score ≥3)	2058 (20.2)	1448 (22.4)	438 (25.2)	44 (3.9)	68 (29.6)	22 (10.0)	11 (6.9)	14 (13.1)	8 (9.9)	5 (9.1)
Anxiety symptoms (GAD-2 score ≥3)	2212 (21.7)	1806 (27.9)	260 (15.0)	48 (4.3)	47 (20.4)	15 (6.8)	6 (3.8)	15 (14.0)	6 (7.4)	9 (16.4)

**Table 2:** Knowledge and attitudes towards COVID-19 vaccination.

Variables	Total *n* = 10183	Brazil *n* = 6470	Malaysia *n* = 1738	Thailand *n* = 1124	Bangladesh *n* = 230	DR Congo *n* = 219	Benin *n* = 159	Uganda *n* = 107	Malawi *n* = 81	Mali *n* = 55
Worry/fear about COVID-19 (Likert score, 1–5)										
Mean ± SD	3.49 ± 1.13	3.71 ± 1.06	3.45 ± 1.06	3.06 ± 1.13	2.70 ± 1.02	2.22 ± 1.10	1.82 ± 0.99	3.34 ± 1.18	2.85 ± 1.21	2.89 ± 1.08
Median (Min, Max)	4 (1, 5)	4 (1, 5)	3 (1, 5)	3 (1, 5)	3 (1, 5)	2 (1, 5)	2 (1, 5)	3 (1, 5)	3 (1, 5)	3 (1, 5)
Participant’s knowledge of COVID-19 vaccine (Yes): n (%)										
Can be reinfected after recovering from COVID-19 infection?	8419 (82.7%)	5535 (85.5%)	1328 (76.4%)	898 (79.9%)	200 (87.0%)	156 (71.2%)	95 (59.7%)	95 (88.8%)	66 (81.5%)	46 (83.6%)
COVID-19 can be prevented by vaccine?	7986 (78.4%)	5822 (90.0%)	951 (54.7%)	721 (64.1%)	116 (50.4%)	129 (58.9%)	89 (56.0%)	86 (80.4%)	39 (48.1%)	33 (60.0%)
There is currently an effective vaccine against COVID-19?	6317 (62%)	4899 (75.7%)	550 (31.6%)	588 (52.3%)	96 (41.7%)	54 (24.7%)	30 (18.9%)	55 (51.4%)	24 (29.6%)	21 (38.2%)
Knowledge about COVID-19 (composite score, 0–3) *										
Mean ± SD	2.23 ± 0.92	2.51 ± 0.78	1.63 ± 0.94	1.96 ± 0.92	1.79 ± 0.92	1.55 ± 0.92	1.35 ± 0.95	2.21 ± 0.83	1.59 ± 0.87	1.82 ± 1.01
Median (Min, Max)	3 (0, 3)	3 (0, 3)	2 (0, 3)	2 (0, 3)	2 (0, 3)	2 (0, 3)	1 (0, 3)	2 (0, 3)	2 (0, 3)	2 (0, 3)
Importance of taking COVID-19 vaccine to protect self (Likert score, 1–5)										
Mean ± SD	4.37 ± 1.01	4.67 ± 0.75	3.90 ± 1.12	4.01 ± 1.06	4.02 ± 0.98	3.11 ± 1.46	2.93 ± 1.41	4.13 ± 1.15	3.01 ± 1.57	3.82 ± 1.23
Median (Min, Max)	4 (1, 5)	5 (1, 5)	4 (1, 5)	4 (1, 5)	4 (1, 5)	4 (1, 5)	3 (1, 5)	4 (1, 5)	3 (1, 5)	4 (1, 5)
Importance of taking COVID-19 vaccine to protect others (Likert score, 1–5)										
Mean ± SD	4.47 ± 0.94	4.75 ± 0.67	4.04 ± 1.06	4.15 ± 0.96	4.12 ± 0.91	3.3 ± 1.49	3.18 ± 1.37	4.30 ± 1.07	3.20 ± 1.62	4.04 ± 1.20
Median (Min, Max)	5 (1, 5)	5 (1, 5)	4 (1, 5)	4 (1, 5)	4 (1, 5)	4 (1, 5)	4 (1, 5)	5 (1, 5)	4 (1, 5)	4 (1, 5)

Note: * Knowledge was the sum of “Yes” (= 1 point) responses to the following questions: (1) possibility of being re-infected after recovering from a previous COVID-19 infection; (2) COVID-19 infection could be prevented by a vaccine; and (3) there is currently an effective vaccine against COVID-19.

**Table 3:** COVID-19 vaccine willingness and attitudes towards vaccination by country (N = 10183).

Participant’s Willingness to Take the COVID-19 Vaccine …	Total *n* = 10183	Brazil *n* = 6470	Malaysia *n* = 1738	Thailand *n* = 1124	Bangladesh *n* = 230	DR Congo *n* = 219	Benin *n* = 159	Uganda *n* = 107	Malawi *n* = 81	Mali *n* = 55
	*n* (%)	*n* (%)	*n* (%)	*n* (%)	*n* (%)	*n* (%)	*n* (%)	*n* (%)	*n* (%)	*n* (%)
At 90% effectiveness	7775 (76.4)	5753 (88.9)	962 (55.4)	658 (58.5)	163 (70.9)	72 (32.9)	36 (22.6)	70 (65.4)	36 (44.4)	25 (45.5)
At 95% effectiveness	9041 (88.8)	6095 (94.2)	1366 (78.6)	981 (87.3)	206 (89.6)	130 (59.4)	77 (48.4)	95 (88.8)	50 (61.7)	41 (74.5)
Possible reasons for refusing to take the COVID-19 vaccine										
I don’t think COVID-19 exists	272 (2.7)	9 (0.1)	18 (1.0)	224 (19.9)	0 (0.0)	8 (3.7)	7 (44)	2 (1.9)	3 (3.7)	1 (1.8)
I think the vaccine is not effective	1540 (15.1)	428 (6.6)	410 (23.6)	500 (44.5)	44 (19.1)	65 (29.7)	37 (23.3)	19 (17.8)	17 (21.0)	20 (36.4)
I think the vaccine is designed to harm us	456 (4.5)	101 (1.6)	142 (8.2)	53 (4.7)	5 (2.2)	44 (20.1)	59 (37.1)	21 (19.6)	23 (28.4)	8 (14.5)
I am scared of side-effects of the vaccine	4198 (41.2)	1775 (27.4)	1287 (74.1)	665 (59.2)	155 (67.4)	107 (48.9)	92 (57.9)	54 (50.5)	34 (42.0)	29 (52.7)
My body is naturally strong, I don’t need a vaccine to fight COVID-19	365 (3.6)	60 (0.9)	101 (5.8)	102 (9.1)	23 (10.0)	37 (16.9)	16 (10.1)	6 (5.6)	14 (17.3)	6 (10.9)
I already had COVID-19, so I think I am immune to the disease	114 (1.1)	79 (1.2)	8 (0.5)	5 (0.4)	14 (6.1)	5 (23)	1 (0.6)	0 (0.0)	1 (1.2)	1 (1.8)
The COVID-19 pandemic is finished in my country, no need for a vaccine now	71 (0.7)	19 (0.3)	12 (0.7)	16 (1.4)	4 (1.7)	6 (2.7)	5 (3.1)	0 (0.0)	6 (7.4)	3 (5.5)
Importance of taking COVID-19 vaccine to protect self										
Strongly disagree	370 (3.6)	111 (1.7)	82 (4.7)	41 (3.7)	6 (2.7)	54 (24.7)	40 (25.2)	6 (5.7)	24 (29.6)	6 (10.9)
Disagree	334 (3.3)	86 (1.4)	119 (6.8)	61 (5.4)	7 (3.0)	21 (9.7)	22 (13.8)	7 (6.5)	9 (11.1)	2 (3.6)
Neutral	831 (8.2)	177 (2.7)	328 (18.9)	199 (17.7)	50 (21.7)	30 (13.7)	27 (17.0)	7 (6.5)	8 (9.9)	5 (9.1)
Agree	2289 (22.5)	1057 (16.3)	575 (33.1)	372 (33.1)	81 (35.2)	74 (33.8)	49 (30.8)	34 (31.8)	22 (27.2)	25(45.5)
Strongly agree	6359 (62.4)	5039 (77.9)	634 (36.5)	451 (40.1)	86 (37.4)	40 (18.3)	21 (13.2)	53 (49.5)	18 (22.2)	17 (30.9)
Importance of taking COVID-19 vaccine to protect others										
Strongly disagree	313 (3.1)	99 (1.5)	66 (3.8)	29 (2.6)	5 (2.2)	49 (22.4)	32 (20.1)	5 (4.7)	23 (28.4)	5 (9.1)
Disagree	221 (2.2)	47 (0.8)	92 (5.3)	34 (3.0)	4 (1.7)	18 (8.2)	15 (9.4)	4 (3.7)	5 (6.2)	2 (3.6)
Neutral	673 (6.6)	119 (1.8)	267 (15.4)	173 (15.4)	41 (17.8)	24 (11.0)	30 (18.9)	7 (6.5)	10 (12.3)	2 (3.7)
Agree	2111 (20.7)	837 (12.9)	589 (33.9)	395 (35.1)	88 (38.3)	75 (34.2)	56 (35.2)	29 (27.1)	19 (23.5)	23 (41.8)
Strongly agree	6865 (67.4)	5368 (83.0)	724 (41.6)	493 (43.9)	92 (40.0)	53 (24.2)	26 (16.4)	62 (57.8)	24 (29.6)	23 (41.8)

Note: Percentages (%) are within country comparisons.

**Table 4:** Cross-tabulation of reasons for vaccine hesitancy with demographic and health status variables.

Variable	I Don’t Think COVID-19 Exists	I Think the Vaccine Is Not Effective	I Think the Vaccine Is Designed to Harm Us	I Am Scared of Side-Effects of the Vaccine	My Body Is Naturally Strong, I Don’t Need a Vaccine to Fight COVID-19	I Already Had COVID-19, so I Think I Am Immune to the Disease	The COVID-19 Pandemic Is Finished in My Country, No Need for a Vaccine Now
	*n* (%)	*n* (%)	*n* (%)	*n* (%)	*n* (%)	*n* (%)	*n* (%)
Gender							
Male	132 (3.1)	918 (21.5)	409 (9.6)	2005 (47.1)	394 (9.2)	120 (2.8)	830 (1.9)
Female	203 (3.4)	1054 (17.8)	343 (5.8)	2978 (52.9)	318 (5.4)	118 (2.0)	64 (1.1)
χ^2^, *p*-value	0.85, *p* = 0.357	22.3, *p* < 0.001	52.5, *p* < 0.001	10.4, *p* = 0.001	52.3, *p* < 0.001	7.4, *p* = 0.007	13.1, *p* < 0.001
Age, years							
18–29	108 (3.4)	651 (20.3)	332 (10.4)	1816 (56.6)	299 (9.3)	91 (2.8)	57 (1.8)
30–39	67 (2.5)	571 (21.1)	201 (7.4)	1462 (54.0)	216 (8.0)	94 (3.5)	37 (1.4)
40–49	76 (4.7)	348 (21.5)	122 (7.5)	700 (43.2)	112 (6.9)	33 (2.0)	41 (2.5)
50–59	75 (5.0)	295 (19.8)	63 (4.2)	634 (42.5)	59 (4.0)	10 (0.7)	8 (0.5)
60 and above	11 (0.9)	107 (9.2)	34 (2.9)	371 (32.0)	27 (2.3)	10 (0.9)	3 (0.3)
χ^2^, *p*-value	49.5, p < 0.001	87.8, *p* < 0.001	96.7, *p* < 0.001	281.9, *p* < 0.001	90.6, *p* < 0.001	48.6, *p* < 0.001	36.4, *p* < 0.001
Highest education level attained							
Primary/Secondary	77 (7.1)	173 (16.0)	61 (5.6)	543 (50.1)	37 (3.4)	13 (1.2)	8 (0.7)
Completed undergraduate degree	195 (4.2)	1043 (22.3)	453 (9.7)	2546 (54.5)	469 (10.0)	150 (3.2)	98 (2.1)
Completed postgraduate degree	63 (1.4)	756 (17.1)	238 (5.4)	1895 (42.8)	205 (4.6)	75 (1.7)	41 (0.9)
χ^2^, *p*-value	109.7, *p* < 0.001	49.6, *p* < 0.001	67.8, *p* < 0.001	126.3, *p* < 0.001	126.6, *p* < 0.001	29.9, *p* < 0.001	26.3, *p* < 0.001
Socioeconomic category							
Low	88 (11.1)	200 (25.2)	92 (11.6)	415 (52.3)	72 (9.1)	5 (0.6)	21 (2.6)
Lower middle	192 (4.0)	1057 (22.3)	460 (9.7)	2497 (52.6)	340 (7.2)	95 (2.0)	64 (1.3)
Upper middle	45 (1.1)	664 (15.7)	174 (4.1)	1947 (46.0)	273 (6.4)	121 (2.9)	45 (1.1)
High	11 (2.7)	51 (12.5)	26 (6.4)	125 (30.6)	28 (6.9)	16 (3.9)	17 (4.2)
χ^2^, *p*-value	226.4, *p* < 0.001	92.6, *p* < 0.001	125.0, *p* < 0.001	99.3, *p* < 0.001	7.5, *p* = 0.057	22.0, *p* < 0.001	34.0, *p* < 0.001
Residential setting							
Rural	137 (17.3)	243 (30.7)	60 (7.6)	419 (53.0)	76 (9.6)	5 (0.6)	11 (1.4)
Suburban/Slum	45 (3.4)	264 (20.1)	124 (9.5)	656 (50.0)	138 (10.5)	38 (2.9)	19 (1.4)
Urban	154 (1.9)	1466 (18.1)	569 (7.0)	3908 (48.4)	498 (6.2)	194 (2.4)	116 (1.4)
χ^2^, *p*-value	537.5, *p* < 0.001	73.6, *p* < 0.001	9.7, *p* = 0.008	7.0, *p* = 0.030	42.2, *p* < 0.001	12.1, *p* = 0.002	0.01, *p* = 0.994
Student or worker in the health sector							
Yes	192 (4.0)	1017 (21.0)	374 (7.7)	2492 (51.4)	378 (7.8)	136 (2.8)	85 (1.8)
No	143 (2.7)	956 (17.9)	378 (7.1)	2491 (46.7)	334 (6.3)	102 (1.9)	62 (1.2)
χ^2^, *p*-value	13.1, *p* < 0.001	15.3, *p* < 0.001	1.5, *p* = 0.223	22.7, *p* < 0.001	9.3, *p* = 0.002	8.9, *p* = 0.003	6.3, *p* = 0.012
COVID-19 testing/Infection status							
Not tested/Does not know test results	292 (4.7)	1392 (22.3)	524 (8.4)	3166 (50.6)	486 (4.8)	64 (1.1)	85 (1.4)
Tested, but negative	37 (1.2)	475 (15.6)	181 (5.9)	1371 (45.0)	142 (4.7)	41 (1.3)	21 (0.7)
Tested, but positive	6 (0.4)	105 (12.0)	47 (5.3)	446 (50.8)	85 (9.7)	134 (15.2)	41 (4.7)
χ^2^, *p*-value	97.4, *p* < 0.001	92.3, *p* < 0.001	24.0, *p* < 0.001	27.4, *p* < 0.001	41.1, *p* < 0.001	699.3, *p* < 0.001	76.8, *p* < 0.001
Presence of chronic disease(s)							
Yes	59 (2.4)	452 (18.5)	126 (5.1)	1099 (44.9)	116 (4.7)	76 (3.1)	38 (1.6)
No	276 (3.6)	1520 (19.7)	627 (8.1)	3885 (50.2)	597 (7.7)	162 (2.1)	109 (1.4)
χ^2^, *p*-value	7.8, *p* = 0.005	1.7, *p* = 0.198	23.8, *p* < 0.001	21.2, *p* < 0.001	25.3, *p* < 0.001	8.3, *p* = 0.004	0.268, *p* = 0.605
Depression symptoms (PHQ-2 score ≥3)							
Yes	24 (1.2)	268 (13.6)	92 (4.7)	952 (48.2)	132 (6.7)	93 (4.7)	35 (1.8)
No	311 (3.8)	17.4 (20.8)	661 (8.1)	4032 (49.1)	580 (7.1)	145 (1.8)	112 (1.4)
χ^2^, *p*-value	33.1, *p* < 0.001	52.6, *p* < 0.001	26.7, *p* < 0.001	0.5, *p* = 0.481	0.4, *p* = 0.554	60.5, *p* < 0.001	1.9, *p* = 0.171
Anxiety symptoms (GAD-2 score ≥3)							
Yes	20 (1.1)	238 (12.8)	90 (4.8)	832 (44.8)	104 (5.6)	83 (4.5)	47 (2.5)
No	316 (3.8)	1735 (20.8)	663 (8.0)	4151 (49.9)	608 (7.3)	115 (1.9)	100 (1.2)
χ^2^, *p*-value	35.2, *p* < 0.001	62.5, *p* < 0.001	21.5, *p* < 0.001	15.5, *p* < 0.001	6.8, *p* = 0.009	45.2, *p* < 0.001	18.9, *p* < 0.001

Note: Frequency (n) and percentage (%) are based on “yes” responses to the questions on reasons for vaccine hesitancy.

**Table 5:** Multiple logistic regression models investigating predictors of COVID-19 vaccine willingness at different effectiveness levels (N = 10,183).

Variables	90% Effectiveness ^a^				95% Effectiveness ^b^			
	aOR	95% CI		*p*-Value	aOR	95% CI		*p*-Value
		Upper	Lower			Upper	Lower	
Constant	0.03				0.02			
Age (years)								
18–29	1.49	1.17	1.91	0.001	1.62	1.14	2.28	0.007
30–39	1.33	1.05	1.69	0.017	1.53	1.10	2.15	0.013
40–49	0.95	0.75	1.21	0.680	0.88	0.63	1.23	0.465
50–59	0.98	0.77	1.24	0.859	0.89	0.64	1.25	0.511
60 and above *								
Country								
Brazil *								
Malaysia	0.32	0.25	0.41	<0.001	0.73	0.53	1.00	0.048
Thailand	0.37	0.30	0.45	<0.001	1.54	1.14	2.10	0.006
Bangladesh	0.57	0.47	0.69	<0.001	1.43	1.08	1.90	0.012
African countries†	0.20	0.16	0.24	<0.001	0.51	0.39	0.67	<0.001
Gender								
Male*								
Female	1.00	0.89	1.11	0.938	0.75	0.65	0.88	<0.001
Highest education level attained								
Primary/Secondary *								
Undergraduate	1.48	1.25	1.77	<0.001	1.50	1.19	1.89	0.001
Postgraduate	1.31	1.09	1.58	0.005	1.30	1.02	1.68	0.037
Number of household members	0.99	0.97	1.01	0.249	0.94	0.92	0.97	<0.001
Income status								
Low *								
Lower middle	1.23	1.01	1.49	0.038	1.19	0.92	1.55	0.181
Higher middle	1.75	1.42	2.16	<0.001	1.29	0.98	1.72	0.074
High	1.90	1.32	2.73	<0.001	1.27	0.77	2.08	0.353
Residential setting								
Rural *								
Suburban/Urban slum	1.08	0.86	1.38	0.503	0.98	0.71	1.36	0.924
Urban	0.97	0.79	1.20	0.795	1.04	0.79	1.38	0.768
Student or worker in the health sector								
No *								
Yes	1.11	0.99	1.24	0.080	1.00	0.86	1.17	0.968
COVID-19 testing/Infection status								
Not tested/ Does not know test results *								
Negative	1.35	1.19	1.53	<0.001	1.37	1.15	1.63	<0.001
Positive	1.05	0.86	1.28	0.627	0.92	0.70	1.20	0.536
Presence of chronic disease(s) ^§^								
No *								
Yes	0.81	0.71	0.92	0.001	0.92	0.76	1.11	0.394
Worry about being infected with COVID-19	1.32	1.25	1.38	<0.001	1.30	1.21	1.40	<0.001
Depression symptoms (PHQ-2 score ≥3)								
Screened negative *								
Screened positive	1.06	0.90	1.25	0.503	1.05	0.83	1.34	0.661
Anxiety symptoms (GAD-2 score ≥3)								
Screened negative *								
Screened positive	0.89	0.75	1.06	0.200	0.91	0.70	1.17	0.444
Knowledge of COVID-19 vaccines ^⁋^	2.09	1.96	2.22	<0.001	2.13	1.96	2.31	<0.001
Importance of vaccine to protect self	1.64	1.56	1.73	<0.001	2.49	2.34	2.66	<0.001

Note: ^a^ χ^2^(26) = 3772.37, *p* < 0.001; Nagelkerke R^2^ = 0.43. ^b^ χ^2^(26) = 3400.92, *p* < 0.001; Nagelkerke R^2^ = 0.50. * Reference Group. † Countries in Africa in this study comprised of the Democratic Republic of Congo, Benin, Uganda, Malawi, and Mali. ^§^ Chronic diseases comprised of heart disease, hypertension, diabetes, cancer, HIV, asthma, and tuberculosis. ^⁋^ Knowledge was the sum of “Yes” (=1 point) responses to the following questions: (1) possibility of being re-infected after recovering from a previous COVID-19 infection; (2) COVID-19 infection could be prevented by a vaccine; and (3) there is currently an effective vaccine against COVID-19.

**Table 6:** The COVID-19 situation and the status of the vaccine roll out program in the countries involved in this study.

Country	Total Confirmed Cases as of 9 February 2021 *	Total Deaths as of 9 February 2021 *	Vaccine Roll-Out Date	Vaccine Type	Vaccine Doses Administered as of 9 February 2021 (%) **	Vaccine Doses Administered as of 27 April 2021 (%) **
Brazil	9,524,640	231,534	23 January 2021	Sinovac, AstraZeneca	3.82 million	40.17 million
Malaysia	245,552	896	24 February 2021	Sinovac, Pfizer, AstraZeneca	0	1.37 million
Thailand	23,746	79	28 February 2021	Sinovac, AstraZeneca	0	1.28 million
Bangladesh	538,378	8221	7 February 2021	AstraZeneca	179,318	8.40 million
DR Congo	23,670	681	19 April 2021	AstraZeneca	0	1710
Benin	4193	55	29 March 2021	AstraZeneca Sinovac	0	~70,000 ***
Uganda	39,860	27	10 March 2021	AstraZeneca	0	321,350
Malawi	27,422	874	16 March 2021	AstraZeneca	0	281,049
Mali	8181	339	8 April 2021	AstraZeneca	0	49,903

Note: * World Health Organization. WHO Coronavirus (COVID-19) Dashboard; 2021. Available online: https://covid19.who.int/ (accessed on 1 May 2021). ** Our World in Data. Coronavirus (COVID-19) vaccinations; 2021. Available online: https://ourworldindata.org/covid-vaccinations (accessed on 1 May 2021). *** Gouvernement de la République du Bénin. Informations Coronavirus (COVID-19); 2021. Available online: https://www.gouv.bj/coronavirus/ (accessed on 27 April 2021).

## Data Availability

Data are available upon reasonable request. Data are available on the International Consortium (International Citizen Project COVID-19 (ICPcovid): http://www.icpcovid.com) website and could be used by other investigators on request. De-identified participant data are available.
